# Wearable Technology for Monitoring Electrocardiograms (ECGs) in Adults: A Scoping Review

**DOI:** 10.3390/s24041318

**Published:** 2024-02-18

**Authors:** Ekta Singh Dahiya, Anubha Manju Kalra, Andrew Lowe, Gautam Anand

**Affiliations:** Institute of Biomedical Technologies (IBTec), Auckland University of Technology, Auckland 1010, New Zealand; ekta.dahiya@aut.ac.nz (E.S.D.); andrew.lowe@aut.ac.nz (A.L.); gautam.anand@aut.ac.nz (G.A.)

**Keywords:** electrocardiography, wearables, electronic devices

## Abstract

In the rapidly evolving landscape of continuous electrocardiogram (ECG) monitoring systems, there is a heightened demand for non-invasive sensors capable of measuring ECGs and detecting heart rate variability (HRV) in diverse populations, ranging from cardiovascular patients to sports enthusiasts. Challenges like device accuracy, patient privacy, signal noise, and long-term safety impede the use of wearable devices in clinical practice. This scoping review aims to assess the performance and safety of novel multi-channel, sensor-based biopotential wearable devices in adults. A comprehensive search strategy was employed on four databases, resulting in 143 records and the inclusion of 12 relevant studies. Most studies focused on healthy adult subjects (*n* = 6), with some examining controlled groups with atrial fibrillation (AF) (*n* = 3), long QT syndrome (*n* = 1), and sleep apnea (*n* = 1). The investigated bio-sensor devices included chest-worn belts (*n* = 2), wrist bands (*n* = 2), adhesive chest strips (*n* = 2), and wearable textile smart clothes (*n* = 4). The primary objective of the included studies was to evaluate device performance in terms of accuracy, signal quality, comparability, and visual assessment of ECGs. Safety findings, reported in five articles, indicated no major side effects for long-term/continuous monitoring, with only minor instances of skin irritation. Looking forward, there are ample opportunities to enhance and test these technologies across various physical activity intensities and clinical conditions.

## 1. Introduction

Cardiovascular diseases (CVDs) cross geographic, socioeconomic, or gender boundaries [1]. Developed and lower-/middle-income countries have a higher prevalence of cardiovascular risk factors, incidences of CVD and stroke, and all-cause mortality [1,2]. Additionally, the 2015 Update on Heart Disease and Stroke Statistics by the American Heart Association (AHA) highlighted that both CVD and stroke are the leading causes of health and economic burden in the US and worldwide. According to the World Health Organization (WHO), CVDs are the primary cause of global mortality, with 17.9 million deaths per year. The reported number of CVD deaths is expected to reach >23.6 million by 2030, up from 17.3 million in 2015 [3]. 

**Figure 1:**
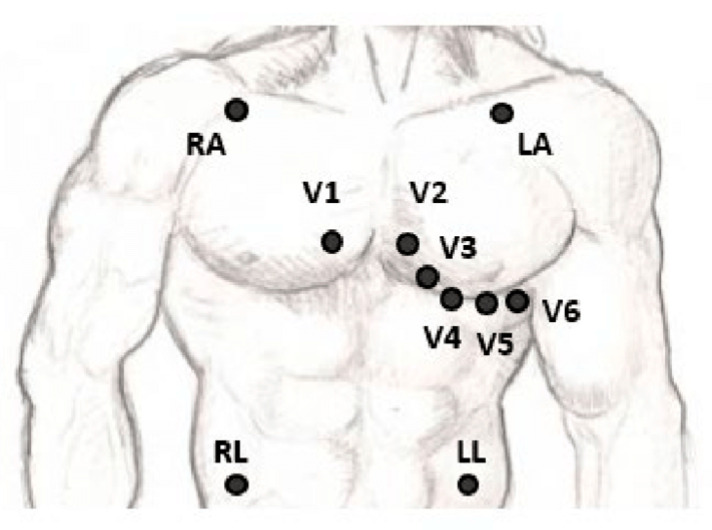
The placement of the leads in a standard 12-lead ECG [5].

Electrocardiograms (ECGs) have become a routine part of any complete medical evaluation and have been used as a diagnostic test since their discovery over 70 years ago. As an ECG provides a waveform showing the electrical activity through the cardiac muscles, many but not all types of damage to the heart tissue can be detected by the ECG [4]. The gold standard 12-lead ECG configuration with its three bipolar limb leads (I, II, and III), three unipolar augmented leads (aVL, aVR, and aVF), six unipolar chest leads (V1–V6), and a reference electrode, as shown in Figure 1, gives spatial information about the cardiac electrical activity [5]. A vital clinical utility of ECG measurement is in detecting acute and chronic myocardial infarction, helping to differentiate coronary artery chest pain from non-cardiac chest pain. Another common diagnostic role is in the identification and management of arrhythmias localizing supraventricular and ventricular arrhythmias [6]. Other cardiovascular diseases, such as myocarditis, pericarditis, and structural deformities, and non-cardiovascular diseases, including hyperthyroidism and hypothyroidism, electrolyte imbalance, and pulmonary embolisms, can manifest as alterations of the ECG curve [7]. 

Currently, ECG monitoring is being used in hospitals (e.g., ICUs, wards, and clinics), homes (telemonitoring, outpatient ambulatory monitoring, and elderly people continuous monitoring at home), and remotely (real-time monitoring, self-diagnosis, and activity monitoring [8]. Some clinical indications for which short intermittent or continuous ECG monitoring has been used are medical drug monitoring, cardiac stress testing, sports performance, fetal ECG, pre-operative assessment, and in operative patients under general anesthesia [9,10,11]. ECG monitoring has been used as a tool for proactive health monitoring by tracking the physiological changes in non-clinical high-stress environments such as deep-sea explorations, wearables in construction, high-altitude environments, and long-duration space exploration missions, with the goal of leveraging ECGs for the early detection of cardiovascular issues and timely intervention [12,13].

**Figure 2:**
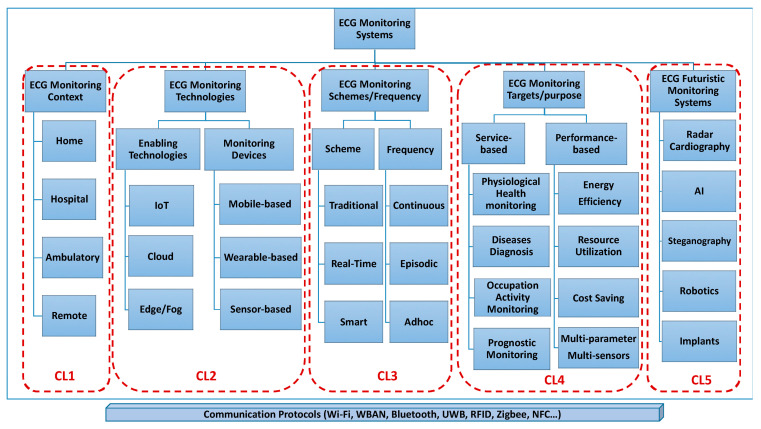
ECG monitoring system clustering (used with permission) [8].

In the past few decades, ECG monitoring systems have been developed, evolved, and are widely used in the healthcare system. ECG monitoring systems are medical devices designed to record and display the electrical activity of the heart over a period of time. There is worldwide demand for a continuous health monitoring system that can detect heart rate variability through which cardiovascular diseases (accounting for 48% of non-communicable disease deaths, as per 2012 WHO Statistics) can be diagnosed and cured at an early stage [14]. Serhani et al. (2020) defined a taxonomy of ‘clusters’ for ECG monitoring systems (EMSs) as Context-aware EMS, Technology-aware EMS, EMS based on Schemes and Frequency, EMS Targets and Purposes, and Futuristic EMS, as shown in Figure 2. The second cluster of technology-aware individuals emphasized wearable devices integrated within an ambulatory, home, or patient/user setup, providing the means for the wireless monitoring of cardiovascular health [8]. 

Wearable ECG devices could be in the form of an ‘on-body patch’ or a contact-less sensor as a smart watch, ‘textile-base’ vest, or capacitive sensors integrated within patients’ stretchers, beds, and wheelchairs [8]. Regardless of the type of sensor, these EMSs integrate with the device to record and retrieve the ECG signals and conduct processing to present a trackable outcome. Prieto-Avalos et al. (2022) reviewed the commercial and non-commercial wearable devices for the physical monitoring of the heart, and they concluded that the majority of such devices have ECG monitoring capacity along with other data; however, improvement in the user’s health is limited without healthy personal habits [15]. Table 1 categorizes these wearable devices with ECG monitoring capacity using either single-lead or multi-lead continuous or real-time ECG. Heart rate during rest and activity can be calculated through the ECG or photoplethysmography (PPG) sensors by calculating beat-to-beat time intervals. 

The wearable wireless ECG devices are designed as a system of electrodes, an analog front-end (AFE), a data acquisition (DAQ) system, a digital signal processing (DSP) unit, wireless communication technology such as Bluetooth, IR, WiFi, and power consumption [8,17,18]. Although many ambulatory ECG monitoring systems have been commercialized to date, a major problem is still faced due to patients/athletes performing motion-related activities that introduce unwanted signal noise that makes monitoring less effective [19]. The frequency spectrum of the motion artifact overlaps the ECG; therefore, it is the most difficult form of noise to be removed [20]. A recent systematic review discussed the challenges of the present monitoring systems, which are rich in diversity and variability. The key challenges identified were manual static screening, the need to learn device operations at the user’s end, the effect on signal quality during real-time long-term monitoring, data processing, analysis and interpretation for the amount of data generated, sensor type and size and designs to keep it user-friendly, and being biocompatible for long-term monitoring [8,17]. Moreover, advances in mobile operating systems and the emergence of artificial intelligence bring their own benefits and challenges [15].

A scoping review is the best choice when the research questions are broad-identifying main concepts, theories, and knowledge gaps in a body of literature and systematically reviewing the data qualitatively [18,21]. We chose to go with the scoping review methodology to review and summarize the evidence on the performance and safety of multi-channel, sensor-based biopotential wearable devices in adults, providing direction for our future research work, where ‘performance’ will be defined based on accuracy, signal quality, comparability to the gold standard, visual assessment, and ‘safety’ as any side effect or adverse reaction on short- or long-term monitoring. 

## 2. Methods

This scoping review used the framework provided by Tricco et al. as the Preferred Reporting Items for Systematic Reviews and Meta-Analyses extension for Scoping Reviews (PRISMA-ScR) checklist [21]. We initiated the review process by developing a review protocol stating the objectives and screening strategies. The Methods Section is organized into five steps: (1) identifying the research question; (2) identifying the relevant studies; (3) study selection; (4) charting the data; and (5) clinical data appraisal, collating, summarizing, and reporting results.

### 2.1. Identifying Research Questions

What is the extent of the scientific literature on monitoring ECGs with multichannel biopotential wearable devices in adults?

We sought to gather information based on the following research questions: (i) determine how the new technologies were designed; (ii) are these devices validated against the gold standard (12-lead ECG); and (iii) screen the selected research for performance and safety outcomes.

### 2.2. Identifying the Relevant Studies

The evaluation was carried out to provide a broader search of the existing literature and a comprehensive description of a given theme, allowing for the identification of gaps in scientific knowledge. The literature was assessed to determine whether the evidential data were sufficient for clinical evaluation. A P.I.C.O. strategy was defined as follows:

1. Population: adults (>19 years), all genders, with or without cardiac irregularities.
2. Intervention: Multichannel biopotential wearable device (Datalogger).
3. Context/comparison: 12-lead ECGs or other variants of ECGs.
4. Outcome (endpoints): ECG and heart rate variability (HRV).

**Figure 3:**
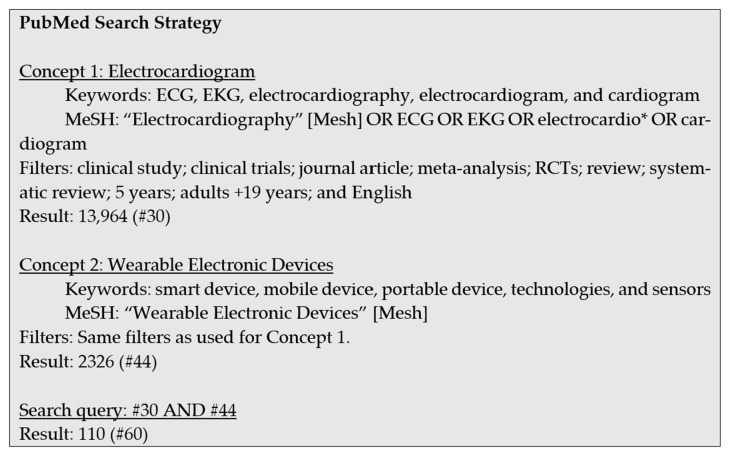
Search strategies for the PubMed database.

The literature search methodology was based on the identified PICO strategy. The search was conducted in January 2021. The search was made from databases such as PubMed, Cochrane, EMBASE, and CINAHL. For each database, concepts were identified, and the possible keywords were included in the search. The search queries were based on the Medical Subject Headings (MeSH) terms and the keywords. The limits or filters were selected to make a concise list of results. The focus was on the past five years, journal articles, and reviews published in English. The search strategy for the PubMed database with the keywords, MeSH terms, and filters is presented in Figure 3 as an example.

### 2.3. Selecting the Studies

We exported records from each database into a master EndNote library and removed duplicates. Articles were selected based on the inclusion and exclusion criteria. The database search retrieved a total of 143 records, and 18 articles were selected that examined the performance and safety of novel concepts of measuring biopotential with a multichannel device and validated them against the gold standard 12-lead ECG or another variant. To have a wider inclusion window, articles/trials with healthy adults as well as those directed towards any cardiovascular condition were selected. Also, commentary and editorials were included if they aligned with the overall objectives. Out of the 18 articles included, three were reviews on wearable devices, which were not included in this scoping review but for a general overview of the current developments. A total of 12 articles (four which were clinical trials) were selected and reviewed in detail. Based on outcomes and the presence of strong evidence, six articles presented the data for device performance only and zero for device safety only (note: some of the literature addressed issues of both performance and safety). The main reasons for the exclusion of articles were duplication, non-availability of full articles, devices working on the principle of optical photoplethysmography (PPG) in smartwatches, or out-of-scope (as defined by the PICO strategy). After retrieving the full-text articles, two authors independently reviewed each paper based on the approved inclusion criteria, and the selected articles were discussed with the team. 

### 2.4. Charting the Data

A summary table abstracting the data from the selected articles was drafted by one researcher. The information recorded in the final extraction included: sample characteristics (size, type of study population), publication year, study location, study design, study objective, type of device, comparative device, and study outcome. Discussion sections from these articles were reviewed to identify their common themes, limitations, and future research directions.

### 2.5. Clinical Data Appraisal, Collating, Summarizing, and Reporting

To ensure a systematic and unbiased appraisal of the data, the researchers set up an appraisal plan that describes the procedure and the criteria to be used for the appraisal. Table 2 shows appraisal criteria based on suitability and data contribution. Based on the defined appraisal plan, the selected articles were graded and categorized as to whether the data addressed the performance or safety of the device in question. The data were then ranked according to the importance of their contribution to establishing the safety and performance of the device and any specific claims about performance or safety. Grades 1–4 were assigned to evidence on both performance and safety, evidence only on performance, evidence only on safety, and no evidence on either parameter, respectively. The summary of the included articles was collated and summarized to report the study characteristics, different study designs, new technologies, and identified themes.

## 3. Results

**Figure 4:**
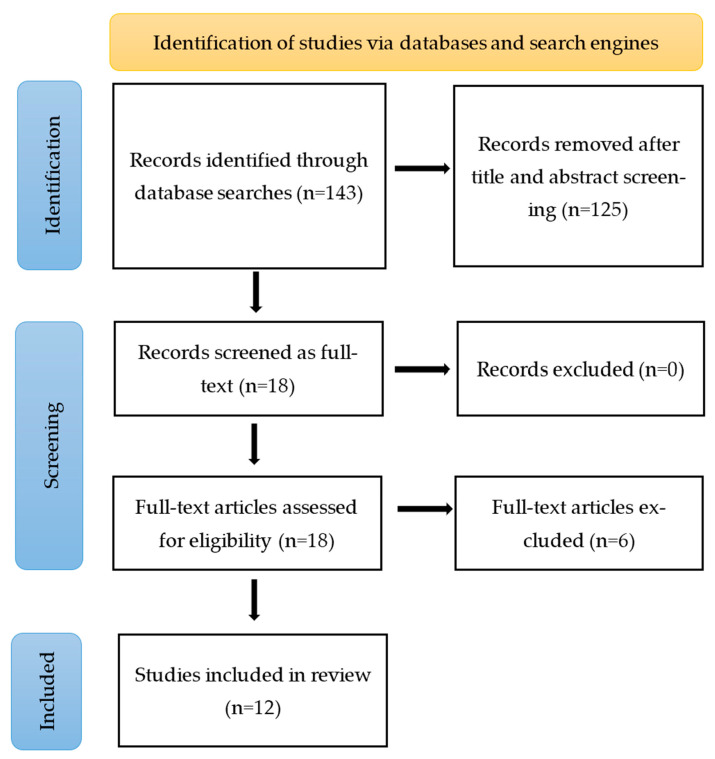
PRISMA flow diagram of the search strategy.

Figure 4 introduces the PRISMA-based flowchart search strategy and the outcome. It includes the number of records excluded after the title and abstract screening, the number of articles retrieved as full-text and excluded after reviewing, and the total number of studies included in the scoping review. We have searched four databases—PubMed, Cochrane, EMBASE, and CINAHL. Removing 125 articles after the title and abstract review left us with 18 full-text articles to review as per the eligibility criteria. At this point, 12 articles met the criteria and were added to this scoping review.

### 3.1. Study Characteristics 

The included studies were published between 2018 and 2020 and were spread across the Pacific, Asian, and European regions. The main locations were the USA (*n* = 3), Taiwan (*n* = 2), Italy (*n* = 2), and one each for Switzerland, Canada, China, Japan, and Brazil. The majority of studies included healthy adult subjects (*n* = 6), while others had healthy control to compare with the patients with atrial fibrillation (*n* = 3), long QT syndrome (LQTS) (*n* = 1), and sleep apnea (*n* = 1). One of the articles was a proof-of-concept for a multi-channel mechanocardiogram (MCG/ECG) to predict left ventricular ejection fraction (LVEF) (W. Y. Lin et al., 2018) [22]. The classification was established based on a variety of biosensing devices utilized for ECG measurement, encompassing all devices utilizing available sensing technologies such as chest-worn (dry-sensing), adhesive (gel-sensing), and textile-based (capacitive-sensing) options. The selected 12 articles investigated different bio-sensor devices categorized as chest-worn belts (Polar H10, Zephyr™ BioHarness™, BLE HR monitor), wrist bands (Kardia Band), adhesive chest strips (BodyGuardian™, Zio Patch), and wearable textile smart clothes (multi-channel mechanocardiogram (MCG)/ECG smart clothes, wearable textile ECG-belt, Omsignal system garment, textile nano-fibers coated with poly (3, 4-ethylene dioxythiophene) (PEDOT)-poly(styrene sulfonate) (PSS) polymer [23,24,25,26,27,28,29,30,31,32,33].

### 3.2. Appraisal of the Clinical Data

Based on the defined appraisal plan shown in Table 2, the selected articles were graded and categorized to determine whether the data addressed the performance or safety of the device in question. Table 3 presents the appraisal grading for the selected articles. As the PICO search strategy included all populations healthy and with any health conditions, most of these studies had appropriate population/patient groups. The data were rated as of high quality with an appropriate study design. The outcomes were aligned with the intended performance of the device. Four out of twelve studies did study long-term monitoring. The data provided were statistically analyzed and sufficient to compare and validate the devices with their respective controls/gold standards. As the devices were not aimed to induce any treatment effect clinically, the papers were graded as C3 (not applicable).

### 3.3. Evaluation of Performance Outcomes

The majority of the selected articles (*n* = 10) had the objective of evaluating the performance of the device under investigation. The two articles that did not account for these were assessing the applicability of a mobile application. The devices (chest straps) that these articles included were commercially available and validated against the gold standard. The purpose of including them in the report was to get an insight into different aspects of the project, such as Bluetooth connection and processing. The remaining 10 articles were ranked based on the similarity in the technology, material, and functions used for the current investigational device, i.e., the Datalogger.

From the ranking given in Table 4, the most important articles that contributed to the demonstration of the overall performance of the device are those with ranks 1–5 [29,31,32,34]. The evidence provided in papers on performance ranged from several outcomes, such as accuracy, signal quality, comparability, and visual assessment of ECG measurements by clinicians/electrophysiologists was sufficient. The statistical analysis performed on the data recorded with the devices evaluated has answered the respective objectives. These reviewed papers can serve as an important baseline to guide the extent of validation and test experiments to be conducted for evaluating the performance of the investigational devices in the future.

### 3.4. Evaluation of Safety Outcomes

The safety outcomes involved evaluating for any side effects and adverse effects of the wearable devices, either physical or mechanical, used in short- or long-term settings. A total of five papers provided direct or indirect evidence of safety for the device they investigated; thus, they have been ranked higher. The outcomes were related to long-term and continuous monitoring and/or skin irritation in the case of wearable textiles or patch electrodes. Whether the ECG sensors were made up of textile or silicone, there were no reported side effects for the devices reviewed. Steinberg et al. (2019) reported that the garments used for OM signals had a minimal risk of skin irritation compared to conventional Ag/AgCl electrodes (7% vs. 47%) [31]. The rest of the included paper did not assess safety parameters as an objective, mainly because the device was worn for a short period or because the device in question has been validated and assessed previously as supported by the literature. Some of these reviewed devices were commercially available and, therefore, have undergone extensive investigation for safety. A detailed summary of these articles is given in Table 4.

## 4. Discussion

The studies under scrutiny exhibited a diverse array of participant profiles and research objectives. While six studies focused on healthy adult subjects, others introduced controlled groups to compare individuals with specific conditions such as atrial fibrillation (AF), long QT syndrome, and sleep apnea. Notably, one article presented a proof-of-concept for a multi-channel mechanocardiogram aimed at predicting left ventricular ejection fraction (LVEF). This diversity underscores the evolving landscape of ECG monitoring and its application in various clinical contexts. The examined bio-sensor devices showcased a range of wearable forms, including chest-worn belts, wrist bands, adhesive chest strips, and innovative wearable textile smart clothes. The different types of wearable device approaches reflect the ongoing efforts to enhance user comfort, increase accessibility, and integrate ECG monitoring seamlessly into individuals’ daily lives. As technology continues to evolve, it opens avenues for novel wearable designs that can cater to specific user preferences and clinical requirements. 

Interestingly, the evidence regarding safety considerations emerged from only five articles among those reviewed. Nonetheless, a noteworthy finding is that regardless of the type of ECG sensor employed, none of the studies reported any adverse effects associated with long-term or continuous monitoring. This is an encouraging indication that wearable ECG technology appears to be well-tolerated by users, at least within the scope of the studies analyzed. While only a limited number of articles addressed safety considerations, the collective evidence indicated a reassuring trend.

The WHO has indicated that more than 25% of adults do not meet the criteria for being physically active and are at a 20% to 30% higher risk of adult mortality [35]. The introduction of activity-tracing wearable devices has promoted physical activity and has been received with great enthusiasm by consumers and the research community. However, this comes with caution as these devices are not medical devices, and very few have only been regulated by the Food and Drug Administration and other regulatory authorities. 

Despite the positive findings on clinical performance and safety, it is essential to acknowledge certain limitations in the current state of wearable technology. The reviewed literature provides a foundational understanding of device capabilities and limitations, yet it also reveals areas requiring further investigation for research and development. Several reviews and journal articles have pointed out the inaccuracy of measurements during some activities [23,36]. For instance, while the studies assessed the accuracy of the devices, there remains a need to explore their performance across different levels of physical activity intensities and various clinical conditions. Such investigations are crucial to understand how these factors may impact the quality and reliability of ECG signals, thereby influencing the diagnostic potential of the devices.

### Future Challenges

Furthermore, future challenges in the realm of wearable ECG monitoring systems encompass a multifaceted landscape. With the invention of artificial intelligence and its integration into ECG monitoring and data analysis, its interpretation, security, and sovereignty pose both opportunities and challenges. Striking a balance between continuous long-term monitoring and efficient battery usage without compromising device performance would be imperative. It would be a challenge for the research community, healthcare professionals, regulators, and industries to design an intuitive user interface that is cost-effective and affordable, establish a clinical validation process fostering standards, and adhere to regulatory frameworks for the seamless integration of wearable ECG devices into mainstream healthcare.

## 5. Conclusions

This scoping review contributes a comprehensive understanding of the performance and safety aspects of novel multi-channel, sensor-based biopotential wearable devices for ECG monitoring in adults. The analyzed studies highlight the strides made in device accuracy and signal quality assessment, while also revealing the promising safety profile of these devices. The evolving landscape offers prospects for refining technology, broadening clinical applications, and advancing personalized health management. As wearable ECG technology continues to shape the future of cardiovascular care, collaboration between researchers, clinicians, and industry partners will be instrumental in unlocking its full potential.

## Figures and Tables

**Table 1:** Commercial wearable devices for ECG measurement and their FDA status [15,16].

Company/Brand	Product	FDA Status
**Watches**		
Adidas	miCoach Fit Smart	NA
Apple	Apple Watch series	A
Biobeat	BB-613WP	A
Fitbit	Flex, One, Charge, Sense, Versa, Luxe, Inspire	A
Garmin	Epix Pro, Fenix 7 pro, Venu, Tactix 7	A
Google	Pixle Watch	NA
Huawei	Huawei Watch GT, Ultimate, Huawei Band	NA
Karacus	DIONE, TRITON	NA
Omron	HeartGuide	A
Samsung	Galaxy Watch 3, 4, 5, 6	A
SmartCardia	INYU	NA
Tom Tom	TomTom Spark	NA
Withings	Steel HR, Move, ScanWatch Horizon	A
**Bands/bracelets**		
AliveCor	Kardiaband	A
BIOSTRAP	Armband HRM	NA
Fitbit	Charge 4	A
HEALBE	GoBe3	U
Microsoft	Microsoft Band	NA
MOCACARE	MOC cuff	A
Under Armour	UA Band	NA
Visi Mobile	The Visi Mobile System	A
Xiaomi	Mi Smart Band 5	U
**Patches**		
BardyDx	Zio Patch	A
BioTelemetry	Bio Tel Heart	A
Corventis Inc	Nuvant MCT	A
Huinno	MEMO Patch	NA
iRhythm	Zio Patch	A
MediBioSense	MediBio Sense MBS HealthStream	A
Preventice Solutions	BodyGuardian	A
Samsung	S-Patch Ex	A
**Clothes**		
HealthWatch Technologies (smart garments)	Master Caution	A
Hexoskin (smart shirt)	Astroskin	NA
Medtronic (chest strap)	Zephyr	A
Polar (chest strap)	Polar H7 Strap	
Sleeplay (smart sock)	Owlet Smart Sock 3	NA
Spire Health Tag	Spire	NA
Vivometrics (smart shirt)	The LifeShirt System	A
Zoll (vest)	LifeVest	A
**Miscellaneous**		
AliveCor (phone attachment)	KardiaMobile	A
Personal Activity Intelligence (phone attachment)	PAI Health	U
Motiv (ring)	Motiv Ring	NA
Oura (finger ring)	Oura Ring	NA
FreeWavz (smart earphones)	FreeWavz-Blue	U
BioSensive Technologies (earrings)	Joule Earrings	NA
SonoHealth	EK Graph	NA
Jabra (headphones)	Sports Pulse Wireless Headphone	NA

The FDA status is current as of January 2024. A: approved; NA: not Approved; and U: unknown.

**Table 2:** Appraisal plan for suitability.

**Appraisal Criteria for Suitability**		
**Criteria**	**Description**	**Grading System**
Appropriate device	Were the data generated from the device in question?	D1: Actual device
		D2: Equivalent device
		D3: Other device
Appropriate device application	Was the device used for the same intended use?	A1: Same use
		A2: Minor deviation
		A3: Major deviation
Appropriate patient group	Where were the data generated from a patient group that was representative of the intended treatment population and clinician condition?	P1: Applicable
		P2: Limited
		P3: Different population
Appropriate report/data collation	Did the reports or collations of data contain sufficient information to be able to undertake a rational and objective assessment?	R1: High quality
		R2: Minor deficiencies
		R3: Insufficient information
**Appraisal Criteria for Data Contribution**		
**Criteria**	**Description**	**Grading System**
Data source type	Was the design of the study appropriate?	T1: Yes
		T2: No
Outcome measures	Did the outcome measures report reflect the intended performance of the device?	O1: Yes
		O: No
Long-term monitoring	Was the duration of monitoring long enough to assess the duration of the treatment’s effects and identify complications?	L1: Yes
		L2: No
		L3: NA/other studies
Statistical significance	Was a statistical analysis of the data provided and was it appropriate?	S1: Yes
		S2: No
Clinical significance	Was the magnitude of the treatment effect observed clinically significant?	C1: Yes
		C2: No
		C3: NA

**Table 3:** Appraisal grading for the selected articles as per the appraisal plan.

References	Appraisal Grading
Boudreaux et al., 2018 [23]	D2/A1/P1/R1/T1/O1/L3/S1/C3
Bumgarner et al., 2018 [24]	D2/A1/P3/R1/T1/O1/L3/S1/C3
Castelletti et al., 2018 [25]	D2/A1/P1/R1/T1/O1/L3/S1/C3
I. M. Lin, 2018 [28]	D2/A1/P1/R1/T1/O1/L3/S1/C3
W. Y. Lin et al., 2018 [22]	D2/A1/P1/R1/T1/O1/L3/S1/C3
Zhang et al., 2018 [33]	D2/A1/P1/R1/T1/O1/L1/S1/C3
Fontana et al., 2019 [34]	D2/A2/P1/R1/T1/O1/L1/S1/C3
Peng et al., 2019 [29]	D2/A1/P1/R1/T1/O1/L3/S1/C3
Reverberi et al., 2019 [30]	D2/A2/P1/R1/T1/O1/L3/S1/C3
Steinberg et al., 2019 [31]	D2/A1/P1/R1/T1/O1/L1/S1/C3
Tsukada et al., 2019 [32]	D2/A1/P1/R1/T1/O1/L1/S1/C3
Pereira, Alves, Silva, Costa, and Silva, 2020 [26]	D2/A2/P1/R1/T1/O1/L3/S1/C3

Appropriate device—D2: equivalent device; appropriate device application—A1: same use, A2: minor deviation; appropriate patient group—P1: applicable, P3: different population; appropriate report/data collection—R1: high quality; data source type—T1: yes; outcome measures—O1: yes, long-term monitoring—L1: yes, L3: NA/other studies; statistical significance—S1: yes, clinical significance—C3: NA. For further details, refer to Table 2.

**Table 4:** Summary of the included articles.

References	Wearable Devices	Number of Participants (N), Group, and Methodology	Results	Conclusion	Comment on Performance and Safety
Fontana et al., 2019 [34]	A wearable textile ECG belt compared against lab polysomnography (PSG).	N = 12 patients with sleep apnea, aged 48–59; BMI: 28–35.5; sleep monitoring for 28 nights at home; measurements compared to clinical data.	Artifact percent: home (9.7% +/− 14.7%) and clinical (7.5% +/− 10.8%); comparable SNR in both settings.	Textile ECG belt: home vs. clinical signal quality comparison: no reduction; signal quality improved compared to clinical PSG.	Long-term monitoring; no adverse effects reported. Grade: 1 Rank: 1
Steinberg et al., 2019 [31]	OM signal system: single-lead wearable ECG sensors vs. three-lead ECG Holter.	N = 15 healthy subjects; garment with three silicone electrodes recorded single-lead ECG for 24 h; signal quality assessed by three electrophysiologists for PQRST distinction.	Signal quality and accuracy matched Holter monitoring (84% vs. 93% electrophysiologists rating, *p* = 0.06); Noise level comparable to Holter recording.	OM garments (shirt and bra): novel wearable ECG sensors; rich signals for rhythm analysis; ease of use, validated against standard Holter recordings.	Evidence on performance and safety in long-term monitoring provided. Grade: 1 Rank: 2
Tsukada et al., 2019 [32]	Conductive textile vs. single-lead ECG Holter system.	N = 66 healthy adults tested textile electrode pads in sportswear for comfort, conductivity. Motion artifacts and noise compared with conventional electrodes.	PQRST patterns same. Conventional electrodes: louder signal. Twisting: noise. Textile stays conductive after 50 washes.	Single-lead textile electrodes in inner garment: feasible for continuous ECG monitoring, except during vigorous trunk movement.	No skin irritation reported. Article meets performance and safety criteria. Grade: 1 Rank: 3
Peng et al., 2019 [29]	Active electrode-based ECG with flexible materials: textile, copper tape, and flexible circuit. Passive Ag/AgCl electrodes used for validation.	N = 10 healthy subjects; created hardware for active electrodes, signal processing, and data transmission; Measured ECG through clothes; evaluated quality in three postures.	Effective, clear ECG waves with all materials; FPC best quality signals (*p* < 0.05); supine position best signals due to good contact; side lying worst quality.	Detects R waves accurately; calculated SNR compares material quality, not true SNR; FPC material produces clear PQRST waves in sitting and supine positions.	Active electrode ECG system tested with Ag/AgCl electrodes; safety not checked; no bad effects with non-contact sensor. Grade: 2 Rank: 4
Zhang et al., 2018 [33]	Zio patch (Zio).	N = 45; patients aged 65+ with AF; group 1 self-applied; group 2 in-office; ECG signals measured; wear time compared.	No difference in the mean wear time (*p* = 0.76) between groups; skin irritation most common adverse reaction (N = 3); self-application equivalent to in-office application.	Zio’s small, leadless, self-contained form with easy installation ensures high self-application success; smaller sample size limited understanding of ethnicity and other patient factors.	Editorial version of feasibility study covers performance and safety; small sample size limits applicability. Grade 2 Rank: 5
W. Y. Lin et al., 2018 [22]	Multi-channel MCG/ECG compared to single-lead ECG bio-amplifier.	N = 48; framework designed for MCG/ECG data acquisition; implemented as wearable smart clothing for cardiac monitoring; usability study conducted (N = 48, age > 20 years) to understand users’ behavioral intention.	ECG from monitoring circuit comparable to Bio Amp ECG with standard electrodes; validation of MCGs and ECG smart monitoring clothes.	Unique, validated smart clothes for cardiac health monitoring designed; capacity for long-term, continuous monitoring; integrated with smart clothing for real-time data analysis.	Wearable device: research prototype; paper covers design, development, processing, assessment; validated in patients. Grade: 1 Rank: 6
I. M. Lin, 2018 [28]	CRST: Zephyr BioHarness chest belt + Bluetooth/mobile; RT: MioAlpha HR watch + Bluetooth/mobile; both compared to ProComp Infiniti biofeedback device EC.	N = 96; healthy adults: CRST, RT, and control; study: psychological questionnaires (depression and anxiety); pre and post-test: ECG, EEG, and breathing rates; CRST: pace breathing; RT: muscle relaxation.	CRST group: higher HRV, lower breathing rates post-test than RT, C groups; no significant EEG effect pre- and post-test in all groups.	The use of a CRST mobile application increased balance in the autonomic nervous system at the resting state.	Zephyr BioHarness compared to ECG; objective: compare training programs; no safety assessment evidence. Grade: 2; rank: 7
Castelletti et al., 2018 [25]	BodyGuardian™ (BG) unit made of electrode gel, sensor, and adhesive layer. This was compared against a 12-lead Holter ECG.	N = 36; healthy and LQTS patients; validation study; Bland–Altman plot compared remote automated QTc (BGM) with manual monitoring (MM).	In all 36 subjects, QTc: MM 446 ± 41 ms, BGM 445 ±47 ms; mean ± SE BAp for QTc: all subjects −1.4 ± 1.8 ms, controls 8.3 ± 2.3 ms, LQTS −7.2 ± 2.5 ms; disagreement < 15 ms: all subjects, controls, LQTS 57%, 63%, and 54%.	This wearable monitoring system reliably identifies a prolonged QT interval and probably also subjects at risk for drug-induced LQTS.	Article compared new automated system reliability; suggested application for identifying drug-induced LQTS; no focus on BG device safety. Grade: 2; rank: 8
Bumgarner et al., 2018 [24]	Kardia Band (KB) used with an Apple watch recorded single-channel ECG. This was compared against a 12-lead ECG.	N = 100; AF patients for cardioversion; observational/validation study; pre-CV ECG, KB recording; post-CV ECG, KB recording if CV; sensitivity, specificity, and K coefficient compared with ECG; physician interpretation.	Compared with ECG, the KB interpreted AF with 93% sensitivity, 84% specificity, and a K coefficient of 0.77.	The KB algorithm for AF detection supported by physician review can accurately differentiate AF from sinus rhythm.	Tested for sinus vs. atrial fibrillation; high sensitivity, specificity; discusses safe, durable platform for recording review, storage; FDA-approved, safety reviewed. Grade: 2; rank: 9
Boudreaux et al., 2018 [23]	Eight wearable devices compared to the gold standard for HR (6-lead ECG) and metabolic analyzer for EE; devices: AWS2, FB, FC2, GVHR, TT, PA360, PH7, and BSP headphones.	N = 50; healthy subjects (age: 18–35); validation study; graded cycling trials; three sets of four resistance exercises at 10-rep max loads; HR, EE recorded; validity established with MAPE ≤ 10%.	Polar H7, BSP valid for both exercise modes (cycling: MAPE = 6.87%, R = 0.79; resistance: MAPE = 6.31%, R = 0.83); Apple Watch Series 2 most valid for cycling (MAPE = 4.14%, R = 0.80); BSP most accurate for resistance exercise (MAPE = 6.24%, R = 0.86); no device valid for EE in any exercise.	Across all devices, as the exercise intensity increased, there was a greater underestimation of HR. EE estimation was inaccurate during cycling or resistance exercise.	Most devices use PPG principle (*n* = 7); polar H7 focused in review; polar H7 validated against standard ECG, no significant difference; paper lacks specific safety mention. Grade: 2 Rank: 10
Pereira et al., 2020 [26]	Heart rate variability (HRV) Expert (CardioMood) smartphone app connected with a chest strap (Polar H10) was compared against a 5-lead conventional ECG.	N = 31; Healthy male runners (mean age: 36.1 ± 6.3); RR intervals recorded by smartphone app, conventional ECG for 5 min; HRV assessed supine, sitting; time-domain indices, frequency-domain indices, and non-linear indices recorded.	No statistically significant difference in both positions (*p* > 0.05) was observed; a strong correlation coefficient was observed between the heart rate variability indexes and all variables (r = 1.0; *p* = 0.00).	Smartphone app, chest strap provided excellent ECG compliance; all variables in time, frequency domain, nonlinear indices assessed; regardless of position; app replaces ECG for HRV analysis in runners.	The objective was to evaluate the accuracy of the smartphone app. The device used (polar H10) is a validated device; no strong evidence on performance and safety. Grade: 4 Rank: 11
Reverberi et al., 2019 [30]	A wearable chest-strap BT HR monitor combined with RITMIA app was compared against the 12- lead ECG interpreted by the physician.	N = 95; patients with atrial fibrillation; ECG recorded with 12-lead, chest-strap; before, after elective cardioversion (ECV) procedure; two cardiologists reviewed and compared the data; the feasibility, sensitivity, specificity, and K coefficient for RITMIA diagnosis were calculated.	The RITMIA app correctly detected AF with 97% sensitivity, 95.6% specificity, and a K coefficient of 0.93; no discomfort while wearing the chest-belt HR sensor was reported.	The RITIMA app algorithm was very accurate in differentiating AF from sinus rhythm as compared to any other commercial chest-strap ECG monitor.	The objective was to establish the RITIMA app used with a wireless chest strap. The recording was conducted for 10 min that did not assess the performance and safety per se. Grade: 4; rank: 12

The grading ranged from grade 1 to 4 based on the appraisal grading shown in Table 3. The ranking of the selected studies was made considering the grading and the evidence of performance and safety outcomes for the wearable device under question.

## Data Availability

No new data was created.

## References

[1] Deaton C., Froelicher E.S., Wu L.H., Ho C., Shishani K., Jaarsma T. (2011). The global burden of cardiovascular disease. Eur. J. Cardiovasc. Nurs..

[2] Feigin V.L., Krishnamurthi R.V., Barker-Collo S., McPherson K.M., Barber P.A., Parag V., Arroll B., Bennett D.A., Tobias M., Jones A. (2015). 30-year trends in stroke rates and outcome in Auckland, New Zealand (1981–2012): A multi-ethnic population-based series of studies. PLoS ONE.

[3] Mozaffarian D., Benjamin E.J., Go A.S., Arnett D.K., Blaha M.J., Cushman M., De Ferranti S., Després J.-P., Fullerton H.J., Howard V.J. (2015). Heart disease and stroke statistics—2015 update: A report from the American Heart Association. Circulation.

[4] Delano M.K. (2012). A Long Term Wearable Electrocardiogram (ECG) Measurement System. Ph.D. Thesis.

[5] Kalra A., Lowe A., Al-Jumaily A. (2018). Critical review of electrocardiography measurement systems and technology. Meas. Sci. Technol..

[6] Rafie N., Kashou A.H., Noseworthy P.A. (2021). ECG interpretation: Clinical relevance, challenges, and advances. Hearts.

[7] Stracina T., Ronzhina M., Redina R., Novakova M. (2022). Golden standard or obsolete method? Review of ECG applications in clinical and experimental context. Front. Physiol..

[8] Serhani M.A., El Kassabi H.T., Ismail H., Nujum Navaz A. (2020). ECG Monitoring Systems: Review, Architecture, Processes, and Key Challenges. Sensors.

[9] Drew B.J., Califf R.M., Funk M., Kaufman E.S., Krucoff M.W., Laks M.M., Macfarlane P.W., Sommargren C., Swiryn S., Van Hare G.F. (2004). Practice standards for electrocardiographic monitoring in hospital settings: An American Heart Association scientific statement from the Councils on Cardiovascular Nursing, Clinical Cardiology, and Cardiovascular Disease in the Young: Endorsed by the International Society of Computerized Electrocardiology and the American Association of Critical-Care Nurses. Circulation.

[10] Khunti K. (2014). Accurate interpretation of the 12-lead ECG electrode placement: A systematic review. Health Educ. J..

[11] Lilly L.S. (2012). Pathophysiology of Heart Disease: A Collaborative Project of Medical Students and Faculty.

[12] Abuwarda Z., Mostafa K., Oetomo A., Hegazy T., Morita P. (2022). Wearable devices: Cross benefits from healthcare to construction. Autom. Constr..

[13] Vinetti G., Lopomo N.F., Taboni A., Fagoni N., Ferretti G. (2020). The current use of wearable sensors to enhance safety and performance in breath-hold diving: A systematic review. Diving Hyperb. Med..

[14] Hayward J., Chansin G. (2015). Wearable Sensors 2015–2025: Market Forecasts, Technologies, Players.

[15] Prieto-Avalos G., Cruz-Ramos N.A., Alor-Hernández G., Sánchez-Cervantes J.L., Rodríguez-Mazahua L., Guarneros-Nolasco L.R. (2022). Wearable devices for physical monitoring of heart: A review. Biosensors.

[16] Bayoumy K., Gaber M., Elshafeey A., Mhaimeed O., Dineen E.H., Marvel F.A., Martin S.S., Muse E.D., Turakhia M.P., Tarakji K.G. (2021). Smart wearable devices in cardiovascular care: Where we are and how to move forward. Nat. Rev. Cardiol..

[17] Cosoli G., Spinsante S., Scardulla F., D’Acquisto L., Scalise L. (2021). Wireless ECG and cardiac monitoring systems: State of the art, available commercial devices and useful electronic components. Measurement.

[18] Sequeira L., Perrotta S., LaGrassa J., Merikangas K., Kreindler D., Kundur D., Courtney D., Szatmari P., Battaglia M., Strauss J. (2020). Mobile and wearable technology for monitoring depressive symptoms in children and adolescents: A scoping review. J. Affect. Disord..

[19] Yan L., Yoo J., Kim B., Yoo H.-J. (2010). A 0.5-*μ* V_rms_ 12-*μ* W Wirelessly Powered Patch-Type Healthcare Sensor for Wearable Body Sensor Network. IEEE J. Solid-State Circuits.

[20] Kim D.-H., Lu N., Ma R., Kim Y.-S., Kim R.-H., Wang S., Wu J., Won S.M., Tao H., Islam A. (2011). Epidermal electronics. Science.

[21] Tricco A.C., Lillie E., Zarin W., O’Brien K.K., Colquhoun H., Levac D., Moher D., Peters M.D., Horsley T., Weeks L. (2018). PRISMA extension for scoping reviews (PRISMA-ScR): Checklist and explanation. Ann. Intern. Med..

[22] Lin W.Y., Ke H.L., Chou W.C., Chang P.C., Tsai T.H., Lee M.Y. (2018). Realization and Technology Acceptance Test of a Wearable Cardiac Health Monitoring and Early Warning System with Multi-Channel MCGs and ECG. Sensors.

[23] Boudreaux B.D., Hebert E.P., Hollander D.B., Williams B.M., Cormier C.L., Naquin M.R., Gillan W.W., Gusew E.E., Kraemer R.R. (2018). Validity of Wearable Activity Monitors during Cycling and Resistance Exercise. Med. Sci. Sports Exerc..

[24] Bumgarner J.M., Lambert C.T., Hussein A.A., Cantillon D.J., Baranowski B., Wolski K., Lindsay B.D., Wazni O.M., Tarakji K.G. (2018). Smartwatch Algorithm for Automated Detection of Atrial Fibrillation. J. Am. Coll. Cardiol..

[25] Castelletti S., Dagradi F., Goulene K., Danza A.I., Baldi E., Stramba-Badiale M., Schwartz P.J. (2018). A wearable remote monitoring system for the identification of subjects with a prolonged QT interval or at risk for drug-induced long QT syndrome. Int. J. Cardiol..

[26] Pereira R.D.A., Alves J.L.D.B., Silva J.H.D.C., Costa M.D.S., Silva A.S. (2020). Validity of a Smartphone Application and Chest Strap for Recording RR Intervals at Rest in Athletes. Int. J. Sports Physiol. Perform..

[27] Fontana P., Martins N.R.A., Camenzind M., Rossi R.M., Baty F., Boesch M., Schoch O.D., Brutsche M.H., Annaheim S. (2019). Clinical Applicability of a Textile 1-Lead ECG Device for Overnight Monitoring. Sensors.

[28] Lin I.M. (2018). Effects of a cardiorespiratory synchronization training mobile application on heart rate variability and electroencephalography in healthy adults. Int. J. Psychophysiol..

[29] Peng S., Xu K., Chen W. (2019). Comparison of Active Electrode Materials for Non-Contact ECG Measurement. Sensors.

[30] Reverberi C., Rabia G., De Rosa F., Bosi D., Botti A., Benatti G. (2019). The RITMIA™ Smartphone App for Automated Detection of Atrial Fibrillation: Accuracy in Consecutive Patients Undergoing Elective Electrical Cardioversion. BioMed Res. Int..

[31] Steinberg C., Philippon F., Sanchez M., Fortier-Poisson P., O’Hara G., Molin F., Sarrazin J.F., Nault I., Blier L., Roy K. (2019). A Novel Wearable Device for Continuous Ambulatory ECG Recording: Proof of Concept and Assessment of Signal Quality. Biosensors.

[32] Tsukada Y.T., Tokita M., Murata H., Hirasawa Y., Yodogawa K., Iwasaki Y.K., Asai K., Shimizu W., Kasai N., Nakashima H. (2019). Validation of wearable textile electrodes for ECG monitoring. Heart Vessel..

[33] Zhang M.J., Roetker N.S., Folsom A.R., Alonso A., Heckbert S.R., Chen L.Y. (2018). Feasibility of using a leadless patch monitor in community cohort studies: The Multi-ethnic Study of Atherosclerosis. Pacing Clin. Electrophysiol..

[34] Fontana P., Martins N.R.A., Camenzind M., Boesch M., Baty F., Schoch O.D., Brutsche M.H., Rossi R.M., Annaheim S. (2019). Applicability of a Textile ECG-Belt for Unattended Sleep Apnoea Monitoring in a Home Setting. Sensors.

[35] Word Health Organization Physical Activity. https://www.who.int/en/news-room/fact-sheets/detail/physical-activity.

[36] Bai Y., Welk G.J., Nam Y.H., Lee J.A., Lee J.-M., Kim Y., Meier N.F., Dixon P.M. (2016). Comparison of consumer and research monitors under semistructured settings. Med. Sci. Sports Exerc..

